# Differential *SLC1A2* Promoter Methylation in Bipolar Disorder With or Without Addiction

**DOI:** 10.3389/fncel.2017.00217

**Published:** 2017-07-21

**Authors:** Yun-Fang Jia, YuBin Choi, Jennifer R. Ayers-Ringler, Joanna M. Biernacka, Jennifer R. Geske, Daniel R. Lindberg, Susan L. McElroy, Mark A. Frye, Doo-Sup Choi, Marin Veldic

**Affiliations:** ^1^Department of Molecular Pharmacology and Experimental Therapeutics, Mayo Clinic, Rochester MN, United States; ^2^Neurobiology of Disease Program, Mayo Graduate School, Mayo Clinic, Rochester MN, United States; ^3^Department of Psychiatry and Psychology, Mayo Clinic Depression Center, Mayo Clinic, Rochester MN, United States; ^4^Division of Biomedical Statistics and Informatics, Mayo Clinic, Rochester MN, United States; ^5^Lindner Center of HOPE, Mason OH, United States; ^6^Department of Psychiatry and Behavioral Neuroscience, University of Cincinnati College of Medicine, Cincinnati OH, United States

**Keywords:** bipolar disorder, *SLC1A2* (EAAT2), methylation, addiction, biomarkers, glutamate

## Abstract

While downregulation of excitatory amino acid transporter 2 (EAAT2), the main transporter removing glutamate from the synapse, has been recognized in bipolar disorder (BD), the underlying mechanisms of downregulation have not been elucidated. BD is influenced by environmental factors, which may, via epigenetic modulation of gene expression, differentially affect illness presentation. This study thus focused on epigenetic DNA methylation regulation of *SLC1A2*, encoding for EAAT2, in BD with variable environmental influences of addiction. High resolution melting PCR (HRM-PCR) and thymine–adenine (TA) cloning with sequence analysis were conducted to examine methylation of the promoter region of the *SLC1A2*. DNA was isolated from blood samples drawn from BD patients (*N* = 150) with or without addiction to alcohol, nicotine, or food, defined as binge eating, and matched controls (*N* = 32). In comparison to controls, the *SLC1A2* promoter region was hypermethylated in BD without addiction but was hypomethylated in BD with addiction. After adjusting for age and sex, the association of methylation levels with nicotine addiction (*p* = 0.0009) and binge eating (*p* = 0.0002) remained significant. Consistent with HRM-PCR, direct sequencing revealed increased methylation in CpG site 6 in BD, but decreased methylation in three CpG sites (6, 48, 156) in BD with alcohol and nicotine addictions. These results suggest that individual point methylation within the *SLC1A2* promoter region may be modified by exogenous addiction and may have a potential for developing clinically valuable epigenetic biomarkers for BD diagnosis and monitoring.

## Introduction

While the hallmark of bipolar disorder (BD) is recurrent mania/hypomania and major depression, patients with BD often present with comorbid addiction, including alcohol (Frye and Salloum, 2006), nicotine (Frye et al., 2013; Lagerberg et al., 2016), and binge eating, which is closely related to food addiction (McElroy et al., 2013; Schulte et al., 2016). Identifying biomarkers of disease in order to appropriately categorize patients into useful subpopulations linked to shared biological disease characteristics may delineate common mechanisms for disease staging, addiction quantification, or treatment guidance (Angst et al., 2013).

Glutamatergic dysregulation has been consistently reported in BD, with the majority of studies revealing elevated glutamate levels in multiple brain regions (Yuksel and Ongur, 2010). Substance use disorders and eating disorders, commonly comorbid in BD, are themselves associated with glutamatergic dysfunction (Bisaga et al., 2008; Gass and Olive, 2008; Ayers-Ringler et al., 2016). CNS extracellular, and synaptic glutamate is regulated by a family of excitatory amino acid transporters (EAATs) which remove glutamate from the synaptic cleft into the glial cell; the majority (∼90%) of glutamate clearance is performed by astrocytic EAAT2 (Su et al., 2003). Post-mortem human brain study by Rao et al. (2012) showed downregulation of EAAT2 protein and mRNA levels in the cortex of patients with BD but not in schizophrenia when compared with controls. Additionally, a number of *in vivo* studies have demonstrated the association of EAAT2 and glutamate clearance dysregulation to the pathophysiology of BD and addiction (Miguel-Hidalgo et al., 2010; Ruby et al., 2010; Dallaspezia et al., 2012; Poletti et al., 2014). However, underlying mechanisms of EAAT2 dysregulation in BD and addiction is unknown.

Increasing evidence suggest the involvement of heterogeneous epigenetic processes, including nucleotide modifications such as DNA methylation, different types of histone modifications, or non-coding RNAs, in the etiology of psychiatric disorders (Pena et al., 2014). Approaches to study epigenetic signatures in humans involve both studies of post-mortem brains but also peripheral tissues (blood and saliva) (Teroganova et al., 2016). In comparison to other major psychiatric disorders (i.e., schizophrenia and major depressive disorder) epigenetic contributions to various BD phenotypes remains largely understudied. DNA methylation is an epigenetic modification that can provide a powerful regulatory mechanism for controlling local transcriptional activity. The presence of CpG dinucleotide repeats, frequently in the promoter region of coding genes in close proximity to the transcription start site, can coordinate the transcriptional repression of the downstream coding region (Sweatt et al., 2013). Interestingly, the *SLC1A2* gene, which encodes for EAAT2, is enriched with CpG sites in the promoter region immediately preceding the transcription start codon, making it a prime candidate for transcriptional regulation by DNA methylation (Zschocke et al., 2007). Furthermore, DNA methylation of specific CpG sites within the *SLC1A2* promoter has been shown to negatively correlate with EAAT2 mRNA expression in astrocytes, suggesting that the EAAT2 gene is susceptible to epigenetic transcriptional modulation (i.e., repression) (Zschocke et al., 2007; Yang et al., 2010). Detailed examination of DNA methylation and other epigenetic modifications of genes such as *SLC1A2* in response to addictions comorbid with BD may allow for the discovery of both diagnostic and treatment response biomarkers (Guidotti et al., 2013).

In this study, we utilize high resolution melting polymerase chain reaction (HRM-PCR) and thymine–adenine (TA) cloning with DNA sequencing analysis in order to localize and quantify DNA methylation within the promoter region of the *SLC1A2* gene of BD patients.

## Materials and Methods

### Subject Blood Samples

This study was approved by the Mayo Clinic IRB. Blood samples from subjects with BD I confirmed by structured diagnostic interview SCID (First et al., 2005) were obtained from the Mayo Clinic Individualized Medicine Biobank for Bipolar Disorder (Frye et al., 2015). Blood samples from healthy control patients (CL) who reported no history of serious mental illness and did not meet the criteria for alcohol abuse or dependence (AA) or nicotine dependence (ND) were obtained from the Mayo Clinic Community Biobank (Olson et al., 2013) (**Table 1** and Supplementary Table S1).

**Table 1:** Characteristics of participants from the Mayo Clinic Community Biobank and Mayo Clinic Individualized Biobank for Bipolar Disorder.

	Demographic and clinical characteristics							Potential confounders as univariate predictors of melting temperature				
Variables	CL	BD only	BD+BE	BD+ND	BD+AA+ND	BD+AA	*p*-value	CL	BD	Estimate	Standard error	*p*-value
	*N* = 32	*N* = 30	*N* = 30	*N* = 30	*N* = 30	*N* = 30		*N* = 32	*N* = 150			
Female	56.3	53.3	76.7	53.3	56.7	40	0.1308	56.3	56	0.0399	0.0265	0.1325
Age	37.4 ± 13.4	42.6 ± 15.4	45.2 ± 14.8	35.1 ± 13.1	35.7 ± 10.4	39.6 ± 12.7	0.0236	37.4 ± 13.4	39.2 ± 13.8	0.0004	0.001	0.6767
BMI	–	30.1 ± 6.9	34.8 ± 7.6	30.3 ± 8.0	27.6 ± 6.2	27.7 ± 4.9	0.0006	–	30.2 ± 7.2	-0.0013	0.0023	0.5737
CIRS	–	4.2 ± 2.9	5.7 ± 3.4	3.6 ± 3.3	4.2 ± 4.3	3.7 ± 3.2	0.0857	–	4.3 ± 3.5	0.0006	0.0048	0.9041
Mood instability	–	1.3 ± 1.6	1.8 ± 1.3	1.7 ± 1.5	2.2 ± 1.2	1.3 ± 1.6	0.0744	–	1.7 ± 1.4	0.0262	0.0123	0.0329
Rapid cycling	–	55.2	53.3	43.3	73.3	36.7	0.0532	–	52.4	0.0131	0.0313	0.6753
Increased severity	–	25	40	43.3	53.3	20	0.0487	–	36.5	0.0502	0.0324	0.121
Mixed episodes	–	9.5	12	16.7	15.4	27.3	0.5525	–	16.1	0.0876	0.0478	0.0667
Cycle acceleration	–	24.1	33.3	37.9	33.3	26.7	0.7902	–	31.1	0.0455	0.0338	0.1781
Psychosis	–	39.3	60	34.5	53.3	30	0.0958	–	43.5	0.0291	0.0318	0.3605
Anxiety disorder	–	44.8	50	63.3	66.7	58.6	0.4047	–	56.8	0.0378	0.0314	0.2288
Atypical antipsychotics	–	20	60	56.7	60	30	0.0016	–	45.3	-0.0173	0.0312	0.5795
Lithium	–	43.3	26.7	6.7	36.7	33.3	0.0226	–	29.3	0.0106	0.0341	0.7552
Lamotrigine	–	17.9	17.9	17.9	33.3	10.7	0.2013	–	18.7	0.0337	0.0457	0.4604
Depakote/valproate	–	0	3.3	0	0	0	0.4024	–	0.7	–	–	–
Alcohol use (more than 1 monthly)	–	36.7	34.5	50	75.9	75.9	0.0006	–	53.3	-0.054	0.0313	0.0859
Alcohol use (ordinal: five categories)	–	–	–	–	–	–	–	–	–	-0.018	0.0122	0.1373
Smoked 100 cigarettes	–	41.4	34.5	100	100	51.7	<0.0001	–	65.3	-0.015	0.033	0.6473
Current smoker	–	10.3	3.6	75	72.4	14.3	<0.0001	–	33.3	-0.074	0.0333	0.0272
Early onset	–	27.6	34.5	26.7	22.2	17.2	0.6450	–	25.7	0.190	0.0112	0.2567

*CL, normal control subjects; BD, bipolar disorder; BE, binge eating disorder or bulimia nervosa; ND, nicotine dependence; AA, alcohol abuse or dependence. BMI, body mass index; CIRS, cumulative illness rating scale; early onset, onset of BD before age of 20 years. Age at enrollment, BMI, CIRS, and mood instability index are shown as mean ± standard deviation; remaining variables are shown as a percentage*.

For HRM-PCR analysis BD patients were selected from five subgroups (*N* = 30 per group) based on clinical evaluation: (1) BD without comorbid BE, ND, or AA; (2) BD with comorbid BE but not AA or ND; (3) BD with comorbid ND but not AA or BE; (4) BD with comorbid AA and ND but not BE; (5) BD with comorbid AA but not BE or ND; For TA-cloning BD patients were selected from two subgroups based on clinical evaluation: (1) BD without comorbid BE, ND, or AA (*N* = 9); (2) BD with comorbid AA and ND but not BE (*N* = 10).

### DNA Extraction

Peripheral blood samples have been previously established as a reliable source of DNA for epigenetic studies in humans (Domschke et al., 2014; Sadeh et al., 2016). To extract the DNA we utilized the Autogen Flex Star with Qiagen Flexigene chemistry (Qiagen, Valencia, CA, United States). Samples that did not meet the requirements for automated extraction were extracted using a Gentra Puregene Blood kit (Qiagen). DNA concentration and quality (Abs260/Abs280) was determined using a Nanodrop 2000 Spectrophotometer (Thermo Scientific, Wilmington, DE, United States).

### Bisulfite Modification

Bisulfite conversion of DNA samples was performed using the EpiTect Fast Bisulfite Kit (Qiagen, Valencia, CA, United States) and purified using the QIAquick PCR purification kit (Qiagen). Unmethylated cytosine nucleotides are converted to uracil while methylated cytosines remain unchanged. PCR was performed in a heated-lid thermal cycler as follows: 95°C for 5 min, 60°C for 20 min, 95°C for 5 min, 60°C for 20 min, and hold at 20°C.

### HRM-PCR and Analysis

High resolution melting PCR enables highly sensitive, labor- and cost-efficient single-locus methylation studies on the basis of DNA high-resolution melting technology, which is widely used to measure relative DNA methylation levels in pre-clinical or clinical epigenetic studies (Wojdacz et al., 2008). HRM-PCR was performed on bisulfite-converted DNA using primer sequences that were designed to indiscriminately amplify both methylated and unmethylated bisulfite-converted DNA. The bisulfite-specific PCR primers (Supplementary Table S2) targeting two regions of the CpG island within the proximal promoter region of the *SLC1A2* gene were designed using MethylPrimer Express v1.0 software (Applied Biosystems, Foster City, CA, United States).

Each reaction was carried out using 1 ng/μL bisulfite-converted DNA, HRM Master Mix (Qiagen), and 0.5 μM of each primer in a total reaction volume of 10 μL. HRM-PCR was performed on a CFX96 Real-Time PCR machine (Bio-Rad, Hercules, CA, United States) in triplicate. Enzyme activation was carried out for 5 min at 95°C and followed by 42 cycles of the following steps: 10 s of denaturation at 95°C, 30 s of annealing at 58°C, and 24 s of extension at 72°C. Samples were then warmed to 95°C at +0.1°C per second. Precision Melt AnalysisTM software (Bio-Rad, Hercules, CA, United States) was used to analyze PCR product melting temperatures.

### TA Cloning and Sequencing Analysis

**FIGURE 1:**
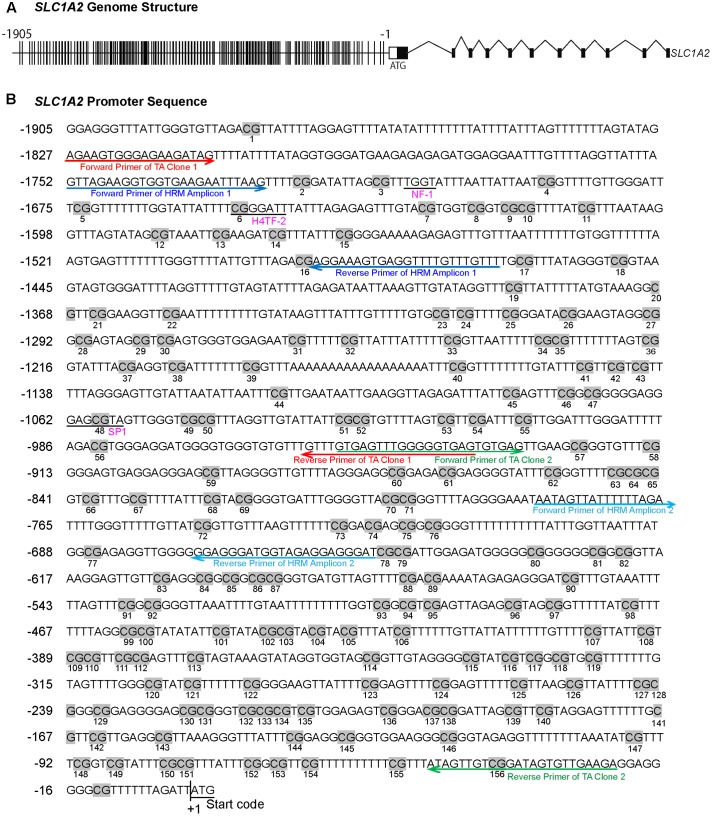
The methylation profile of CpG sites on *SLC1A2* promoter. **(A)** The gene structure profile of *SLC1A2*. Distribution of CpG sites on the proximal *SLC1A2* promoter region approximately 2 kb. The individual lines in the promoter denote each CpG site. **(B)** Distribution of CpG sites, location of HRM and bisulfite-specific primers on the *SLC1A2* promoter region. The number of individual CpG sites is shown in the picture under each CpG site, displayed in gray highlight. The putative promoter elements in the human *SLC1A2* gene are shown in underlined pink text. Arrow indicates primers used in experiments to amplify the interested regions. Red arrow denotes the primers of TA Clone 1; green arrow denotes TA Clone 2; dark blue arrow denotes amplicon 1 in HRM, light blue arrow denotes amplicon 2 in HRM. All primers shown in the picture are bisulfite-specific primers.

Thymine–adenine (TA) cloning and subsequent Sanger sequencing was performed as described previously (Shen and Waterland, 2007; Li and Tollefsbol, 2011) on bisulfite-converted DNA of the CpG island located in the proximal promoter region of the *SLC1A2* gene (**Figure 1**). Due to the substantial length of the CpG island, the region was divided into two segments, generating two TA clones. The bisulfite-specific PCR primers utilized for TA cloning are reported in Supplementary Table S2. We randomly selected 11 CL, 9 BD, and 10 BD with comorbid both AA and ND. The group of BD with comorbid both AA and ND was selected based on hypothesis that having impact of both nicotine and ethanol may have synergistic effect detectable in changes in DNA methylation.

Polymerase chain reaction was performed on the specific promoter region covered by each clone using 500 ng of bisulfite-converted DNA from each subject. Two μL of this PCR product was reclaimed for nested PCR to further amplify the relevant promoter sequences. Both amplification procedures were executed using hot-start PCR with 50 μL reaction volumes containing 10 nM primers, and a master mix containing HotStarTaq polymerase (Qiagen, Catalog No. 203443). The first PCR reaction for both clones was carried out with initial denaturation at 95°C for 15 min, followed by 5 cycles of [2 min at 95°C, 2 min annealing at 64°C and 3 min elongation at 72°C], 35 cycles of [45 s at 95°C, 2 min annealing at 64°C and 1:30 min elongation at 72°C], and a final 10-min extension step at 72°C. The second nested PCR reaction utilized for both clones was performed with initial denaturation at 95°C for 15 min, followed by 5 cycles of [2 min at 95°C, 2 min annealing at 64°C and 3 min elongation at 72°C], and 25 cycles of [45 s at 95°C, 2 min annealing at 64°C and 1:30 min elongation at 72°C], followed by a final 10-min extension step at 72°C.

All PCR products were confirmed on a 1.0% agarose gel. Verified PCR products were cloned into TOPO vectors utilizing the Invitrogen TOPO TA Cloning dual promoter kit (Thermo Fisher Scientific). Two μL of ligation product were used to transfect One Shot *Escherichia coli*, which were subsequently plated on Luria Broth agar plates containing Kanamycin (50 ug/mL) and coated with X-gal. Five to ten white colonies were picked for each subject sample and expanded overnight with Kanamycin selection. Plasmids were purified using the Wizard^®^ *Plus* SV Miniprep DNA Purification System (Promega, A1330) and then Sanger sequenced using the M13 Reverse primer by the Sequencing Core of the Medical Genome Facility at the Mayo Clinic (Rochester, MN, United States). The location and frequency of converted and unconverted cytosine nucleotides within the plasmids of the 5–10 representative colonies selected for each patient sample provided a close approximation of the overall pattern and frequency of CpG methylation of each subject sample.

### Statistical Analysis

One-way analysis of variance (ANOVA) was utilized to examine the overall difference in methylation across the six groups, followed by pairwise *post hoc* tests adjusted for multiple comparisons using Bonferroni’s method for 15 pairwise tests between the six diagnoses groups. A general linear regression model was used to examine the specific effects of BD, ND, AA, and BE as covariates on *SLC1A2* promoter methylation. Unpaired two-tailed Student’s *t*-tests were used to examine the difference between the groups in TA cloning data analysis. *p* < 0.05 was considered statistically significant. Analyses were conducted using SAS (version 9.4; Cary, NC, United States).

## Results

### Patients’ Characteristics

A structured clinical questionnaire was administered by a member of the study team and used to determine historical illness variables (e.g., history of suicide attempts, psychotic symptoms, rapid cycling, and cycle acceleration over time), comorbid psychiatric disorders [including AA, ND, and either binge eating disorder or bulimia nervosa (BE)]. A structured patient questionnaire was completed assessing other relevant clinical and demographic (e.g., age and sex) variables. General medical burden and comorbidity was assessed via the Modified cumulative illness rating scale (CIRS) (Hudon et al., 2005), which measures the severity of patient-reported, organ-specific, comorbid medical illnesses. Overall psychiatric illness burden was evaluated using proxy measures of illness severity including psychosis, mood instability, and comorbid anxiety disorder. Psychosis was evaluated as a dichotomous yes-no variable and deemed present when the patient had a lifetime history of hallucinations or delusions in the absence of plausible medical precipitation. Mood instability was operationalized based upon the lifetime incidence of mixed episodes, rapid cycling, and ultra-rapid/ultradian cycling, as well as cycle acceleration or exacerbation over time (McElroy et al., 2013). Each measure was coded as no = 0 and yes = 1, allowing for mood instability scores ranging from 0 to 5. Anxiety disorder comorbidity was scored 0–6 based upon the lifetime presence of comorbid anxiety disorders including post-traumatic stress disorder, generalized anxiety disorder, social anxiety disorder, obsessive-compulsive disorder, phobia, and panic disorder (**Tables 1**, **2**). Clinical information including BD illness characteristics [body mass index, cumulative illness rating scale, rapid cycling, increased severity, mixed episodes, cycle acceleration, psychosis, anxiety disorder, early onset (<20 years)], treatments (atypical antipsychotics, lithium, valproate, and lamotrigine) and demographic variables (age, sex), current alcohol use (≥2x per month), current smoker status, and lifetime history of smoking ≥ 100 cigarettes were examined as potential confounding univariate predictors of methylation (**Table 1**).

### HRM-PCR Demonstrates Differential Methylation Level of the *SLC1A2* Gene Promoter Region in BD With and Without Addiction

We examined a stretch of approximately 2000 base pairs (2 kb) containing the majority of CpG sites within the *SLC1A2* promoter (**Figure 1A**). HRM-PCR was utilized to specifically interrogate isolated sequences between -1756 and -1465 bp (Amplicon 1) and -782 and -651 bp (Amplicon 2) within the 5′-UTR of *SLC1A2*. These 291 and 131 bp amplicons contain 15 and 6 CpG sites, respectively, representing CpG sites 2–16 and 72–77 of the *SLC1A2* 5′-UTR (**Figure 1B**). Interestingly, subsequent analysis with “signal scan” web software^1^ revealed that amplicon 1 contained well-established consensus sequences for the binding of the transcription factors NF-1 and H4TF-2, which overlapped or existed in close proximity to CpG sites 3 and 6 of the *SLC1A2* 5′-UTR. This software similarly identified an SP-1 binding site centered at CpG 48 located -1059 bp upstream of the transcription start codon within the sequence specified by TA clone 1 (**Figure 1B**).

**FIGURE 2:**
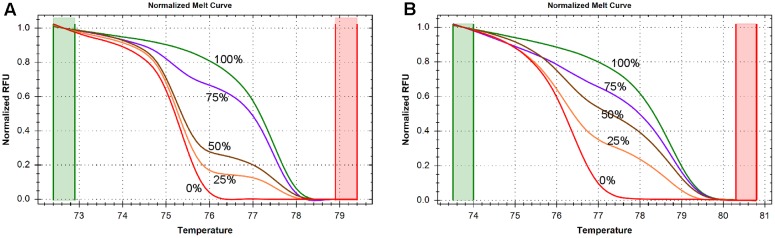
DNA methylation is measured by high resolution melting (HRM) method. **(A)** Normalized melting curves of amplicon 1 between –1759 and –1468. The standards melting curves include 0, 25, 50, 75, and 100% methylated human control DNAs. **(B)** Normalized melting curves of amplicon 2 between –785 and –654. The standards melting curves include 0, 25, 50, 75, and 100% methylated human control DNAs.

In order to determine the accuracy and sensitivity of the *SLC1A2* HRM primers for different quantities of DNA methylation, we assessed the consistency of normalized melting curve derived from the PCR products of human DNA templates containing 0, 25, 50, 75, and 100% methylated cytosine nucleotides using our amplicon 1 and amplicon 2 bisulfite-specific primers (Supplementary Table S2). The normalized melting curves of both amplicon 1 (**Figure 2A**) and amplicon 2 (**Figure 2B**)-primed products displayed finely graded methylation-dependent increases in melting temperature, suggesting that the HRM primers allow for unbiased PCR amplification of both highly and minimally methylated sequences.

**FIGURE 3:**
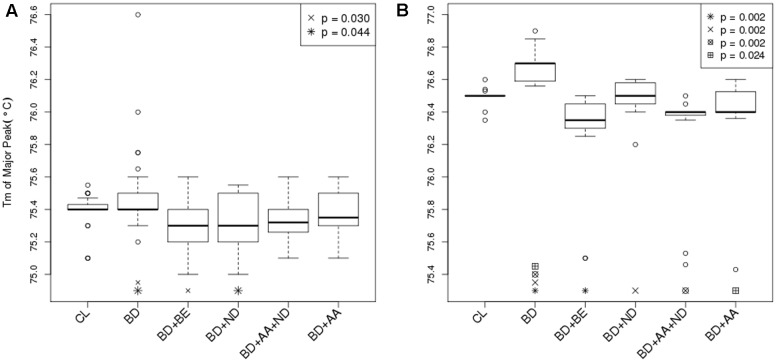
Differential DNA methylation between BD and BD with addiction phenotypes. **(A)** Bar graph displayed Tm of major peak of amplicon 1 between –1759 and –1468 in the 5′ UTR of the *SLC1A2* as reported by HRM-PCR. Healthy control subjects (CL) (*N* = 32); bipolar disorder (BD) without binge eating disorder or bulimia nervosa (BE), nicotine dependence (ND), and alcohol abuse or dependence (AA) (*N* = 30); BD+BE (*N* = 30); BD+ND (*N* = 30); BD+AA+ND (*N* = 30); BD+AA (*N* = 30). **(B)** Bar graph displayed Tm of major peak of amplicon 2 between –785 and –654 in the 5′ UTR of the *SLC1A2* as reported by HRM-PCR. All groups (*N* = 15). Symbols indicate a significant differences compared to BD only group (*p* < 0.05, one-way ANOVA followed by *post hoc* pairwise tests adjusted for multiple comparisons using Bonferroni’s method). Data are expressed as median ± IQR.

The segment of *SLC1A2* promoter region flanked by amplicon 1 (**Figure 3A**) primers was amplified and analysed via HRM-PCR analysis. One-way ANOVA demonstrated significant differences in mean melting temperature (Tm) across the six groups (*p* = 0.0003). *Post hoc* pairwise tests adjusted for multiple comparisons using Bonferroni’s method demonstrated that the reduced melting temperature of samples in patients suffering from BD with BE (*p* = 0.002, corrected *p* = 0.030), and ND (*p* = 0.003, corrected *p* = 0.044) compared to that attained from BD patients without addictions. The BD group with ND and AA addictions also demonstrated lower melting temperature compared to BD, but did not pass the stringent multiple testing threshold (*p* = 0.005, corrected *p* = 0.069). General linear regression modeling suggests that both BE (*p* = 0.0006) and ND (*p* = 0.0014) are characterized by reduced melting temperature, while AA is highly correlated (*p* = 0.0527) with lower melting temperatures (**Table 2**). Using a multivariable generalized linear model adjusted for age and sex, the impact of BE (*p* = 0.0002) and ND (*p* = 0.0009) on Tm remained statistically significant. The melting temperature was significantly higher in females (*p* = 0.0365) and age did not significantly affect melting temperature (**Table 2**).

**Table 2:** Analysis of maximum likelihood parameter estimates using general linear regression model and multivariable generalized linear model.

General linear regression model							Multivariable generalized linear model					
Variables	DF	Estimate	Standard error	95% confidence limits		*p*-value	DF	Estimate	Standard error	95% confidence limits		*p*-value
Intercept	1	75.3884	0.0302	75.3293	75.4475	<0.0001	1	75.3884	0.0302	75.3293	75.4475	<0.0001
Age	–	–	–	–	–	–	1	0.0000	0.0010	-0.0019	0.0000	0.9984
Sex (female)	–	–	–	–	–	–	1	0.0541	0.0259	0.0034	0.1048	0.0365
BD	1	0.0654	0.0405	-0.0139	0.1447	0.1060	1	0.0692	0.0402	-0.0097	0.1481	0.0854
ND	1	-0.0993	0.0311	-0.1604	-0.0383	0.0014	1	-0.1038	0.0313	-0.1652	-0.0424	0.0009
AA	1	-0.0603	0.0311	-0.1214	0.0007	0.0527	1	-0.0576	0.0308	-0.1180	0.0028	0.0616
BE	1	-0.1408	0.0412	-0.2216	-0.0601	0.0006	1	-0.1557	0.0414	-0.2369	-0.0745	0.0002

*BD, bipolar disorder; ND, nicotine dependence; AA, alcohol abuse or dependence; BE, binge eating disorder or bulimia nervosa*.

When demographic and clinical characteristics of the groups used to conduct HRM-PCR analysis (**Table 1**) were compared there was a statistically significant difference between the groups for age (*p* = 0.0236), body mass index (*p* = 0.0006), symptom severity (*p* = 0.0487), atypical antipsychotic use (*p* = 0.0016), lithium use (*p* = 0.0226), current alcohol use (*p* = 0.0006), current smoker status (*p* < 0.0001), and lifetime history of smoking ≥ 100 cigarettes (*p* < 0.0001). Several of these differences were expected as they were inherent to the group design (body mass index for BE; current alcohol use for AA; current smoker status and lifetime history of smoking ≥ 100 cigarettes for ND). In order to address these issues, clinical information including BD illness characteristics [body mass index, cumulative illness rating scale, rapid cycling, increased severity, mixed episodes, cycle acceleration, psychosis, anxiety disorder, early onset (<20 years)], treatments (atypical antipsychotics, lithium, valproate, and lamotrigine) and demographic variables (age, sex), current alcohol use (≥2x per month), current smoker status, and lifetime history of smoking ≥ 100 cigarettes were examined as potential confounding predictors of methylation (**Table 1**). When illness or treatment related variables were considered as univariate predictors of methylation temperature an effect was found for mood instability sum (*p* = 0.0329) and current smoker status (*p* = 0.0272), but not for any other variable (**Table 1**). We note that there was no effect for sex (*p* = 0.1325) in the univariate model, which is different from the modest effect of sex that we observed in a multivariable regression model (*p* = 0.0365) when impact of sex on methylation was analyzed in conjunction with age, BD diagnosis, BE, ND, and AA (**Table 2**).

The segment of *SLC1A2* promoter region flanked by amplicon 2 primers (**Figure 3B**) was amplified and analysed via HRM-PCR analysis. One-way ANOVA across the six groups (*p* < 0.0001), followed by pairwise *post hoc* tests adjusted for multiple comparisons using Bonferroni’s method demonstrated that the Tm major peak of amplification products obtained from BD patients was significantly higher than from CL (*p* < 0.0001, corrected *p* = 0.002). In contrast, reduced melting temperature was shown in samples isolated from patients suffering from BD with addictions [BE (*p* < 0.0001, corrected *p* = 0.002), ND (*p* < 0.0001, corrected *p* = 0.002), AA+ND (*p* = 0.0001, corrected *p* = 0.002), and AA (*p* = 0.002, corrected *p* = 0.024)] compared to that attained from BD patients without addictions. General linear regression modeling describing the relative predictive value of melting temperature for identification of comorbid use disorders suggests that BE (*p* < 0.0001), ND (*p* = 0.011), and AA (*p* < 0.0001) showed reduced melting temperature, while BD showed increased Tm (*p* = 0.035).

Therefore, our data demonstrates that the segments of the promoter region of the *SLC1A2* gene defined by amplicon 2 become hypermethylated in patients with BD alone and hypomethylated in both amplicon 1 and 2 in patients suffering from BD comorbid with BE ND, AA + ND or AA. This suggests that BD and addictions may independently regulate epigenetic modification of the *SLC1A2* promoter.

### TA Cloning and DNA Sequencing Reveal Sequence-Specific Hypermethylation of the *SLC1A2* Promoter in BD and Hypomethylation in BD Patients with Substance Use Comorbidities

**FIGURE 4:**
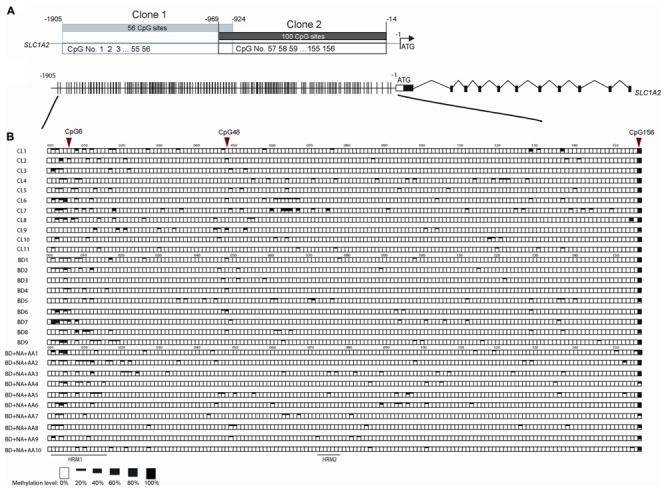
The methylation pattern of individual CpG sites on *SLC1A2* promoter region. **(A)** The profile of two clones in TA cloning and DNA sequencing method, shared 45 bp overlap, covered 156 CpG sites during –1905 to –14 of *SLC1A2* promoter. **(B)** The gene profile of *SLC1A2*. Distribution of CpG sites on the proximal *SLC1A2* promoter. The individual lines in the promoter denote each CpG site (Top panel). Sequences from multiple clones (*n* ≥ 5 from two replicate experiments for each condition) in each subject were examined. The methylation level of individual CpG sites in each subject from three groups including healthy control subjects (CL), bipolar disorder (BD) without alcohol abuse or dependence (AA), nicotine dependence (ND), and binge eating disorder or bulimia nervosa, and BD comorbid with ND and AA are shown in boxes (bottom panel). The average methylation percentages of individual CpG sites are shown in different size of black boxes. The empty box denotes 0% methylation level, while the total black box denotes 100% methylation level.

To further examine the sequence-specific methylation differences within the promoter region of the *SLC1A2* gene of BD patients and BD patients with comorbid ND and AA, we performed TA cloning with subsequent sequencing analysis. This allowed us to fully analyze the 156 CpG sites within the *SLC1A2* promoter from -1905 to -14 bp upstream of the transcription start codon site (**Figure 4A**). The methylation profile examined for each patient in the three relevant groups is shown in **Figure 4B**.

Our previous interrogation of the *SLC1A2* promoter with the online “signal scan” software revealed the presence of three transcription factor binding sites, which overlap about one or more CpG sites within the *SLC1A2* 5′-UTR. These include consensus sequences for the binding of NF-1, H4TF-2, and SP-1, which are located in close proximity to CpG 6 and 48, respectively. These CpG sites are good candidates to carry out important epigenetic regulation of gene expression. We therefore utilized TA cloning with subsequent sequencing to specifically interrogate these sites for differential methylation in patients with isolated BD, BD with ND and AA, and CL.

Statistical analysis of individual CpG sites 6, 48, and 156 revealed significantly altered methylation profiles among groups (**Figure 4B**). Samples from isolated BD exhibited significantly increased methylation at CpG 6 compared to CL (*p* = 0.0246). In contrast, CpG 156 (*p* = 0.0184) methylation level was reduced in BD with AA and ND in comparison to CL. Similarly, CpG sites 6 (*p* = 0.0082) and 48 (*p* = 0.0267) were hypomethylated in BD with AA and ND group in comparison to BD without addiction. CpG site 156 (*p* = 0.0646) trended toward hypomethylation in BD with AA and ND group in comparison to BD without addiction. The analysis for the all CpG sites was shown in Supplementary Table S3.

When demographic and clinical characteristics of the groups used to conduct TA cloning and DNA sequencing analysis (Supplementary Table S1) were compared there was a statistically significant difference between the groups for atypical antipsychotic use (*p* = 0.020), current smoker status (*p* = 0.015), and lifetime history of smoking ≥ 100 cigarettes (*p* = 0.029). Similarly, to the group differences related to smoking observed for larger groups used for HRM-PCR these differences were expected as they are inherent to the group design. Given the small sample size of the groups studied in TA cloning and DNA sequencing analysis we did not control for the impact of the medications so the effect of the use of antipsychotics on the methylation levels cannot be excluded with certainty. However, when the effect of the use of antipsychotics was analyzed as univariate predictors of methylation temperature for larger groups studied in HRM-PCR analysis no statistically significant effect was found (*p* = 0.5795) (**Table 1**).

## Discussion

In this study, we examined the methylation of the *SLC1A2* promoter in DNA from the blood of patients with BD with and without comorbid addiction to alcohol, nicotine, or food. Using HRM-PCR and TA cloning, we demonstrate *SLC1A2* promoter hypermethylation in patients with BD without addiction and hypomethylation in BD with addiction. However, we note that the hypermethylation in BD isolated group compared to CL was only found in amplicon 2 and not in amplicon 1 in HRM-PCR, even though the hypomethylation was found in both amplicons between BD and BD with addictions suggesting a specificity in the function of different regions CpG island. More importantly, these results suggest that examination of the total epigenetic landscape of the *SLC1A2* gene may provide a useful clinical tool for the accurate diagnosis of BD and BD comorbidities and that individual point methylation within the promoter region of a gene encoding for glutamate transporter may be modified by addiction which in a sequence of downstream events could lead to associated functional change in glutamate tone (Zschocke et al., 2007). Further work is encouraged to investigate the role of epigenetic analysis as a component in clinical efforts to develop biomarkers of disease burden in BD.

Bipolar disorder is heterogeneous and complex, producing diverse phenotypes, differential expressivity, and a high level of discordance among identical twins (Labrie et al., 2012). This suggests that non-genetic factors contribute to disease emergence and presentation, including multiple environmental stimuli, which may converge to effect epigenetic regulation and induce stable changes in gene expression (Pena et al., 2014; Kundakovic and Champagne, 2015). Glutamatergic dysfunction plays a prominent role in the development of both BD and addiction. However, the heterogeneity of disease phenotypes and significant discordance among identical twins suggests that epigenetic modulation of the glutamatergic system may contribute to CNS dysfunction in those disorders. Furthermore, recent studies suggest that epigenetic features better reflect disease progression, and may serve as more appropriate biomarkers for the detection or monitoring of disease progression and resolution (Zhubi et al., 2009; Levenson and Melnikov, 2012).

EAAT2 is the principle transporter responsible for glutamate uptake and has recently been implicated in the development of BD and other psychiatric disorders (Lauriat and McInnes, 2007; Rao et al., 2012; Takahashi et al., 2015). To date, there is no epigenetic study that examined EAAT2 in BD patients. However, changes in DNA methylation have been observed at several BD candidate gene loci such as serotonin system genes, brain-derived neurotrophic factor, and catechol-*O*-methyltransferase (Ghadirivasfi et al., 2011; Nohesara et al., 2011; D’Addario et al., 2012) in peripheral samples. The only study, to the best of our knowledge, conducted with peripheral samples of BD patients that reported changes in DNA methylation in the glutamatergic system is the report on increased dystrobrevin binding protein 1 (DTNB1) promoter methylation in BD patients with psychotic depression compared to other BD patients (Abdolmaleky et al., 2015; Fries et al., 2016). The DTNB1, also known as dysbindin, has been suggested to be involved in glutamatergic neurotransmission by influencing exocytotic glutamate release (Domschke et al., 2011). Epigenetic studies of the glutamatergic system in patients with schizophrenia reported differential methylation of the glutamate receptor ionotrophic alpha-amino-3-hydroxy-5-methyl-4-isoxazole propionic acid 2 and 3 and glutamate metabotropic receptors 2, 5, and 8 (Aberg et al., 2012; Kordi-Tamandani et al., 2013; Teroganova et al., 2016). Although no study has yet examined specific changes in histone modifications at genes associated with the glutamatergic system in patients with BD, expression of various histone deacetylases have been found to be significantly altered in peripheral samples of BD patients. For example, expression of histone deacetylase (HDAC) 4 mRNA was increased in BD patients only during depressive episodes, while the expression of HDAC6 and HDAC8 was decreased in both depressive and remissive states compared to controls (Hobara et al., 2010). Fittingly, lymphocytes of BD patients display an active cell and nuclear state characterized by increased ratios of variant histones H3.1, H3.2, H2A.1, H2A.Z, and H2A.X to stereotypical H3 and H2A (Sourlingas et al., 1998) and relaxed de-condensed chromatin ultrastructure (Chrysanthou-Piterou et al., 2009) during depressive and manic episodes. These changes in HDAC activity and chromatin structure may alter the expression of various components of the glutamatergic system known to be under the control of epigenetic regulation. Our data suggests that epigenetic modification of the *SLC1A2* promoter may modulate the expression of EAAT2. This is supported by other studies which demonstrated that *SLC1A2* promoter demethylation and histone hyperacetylation produce robust activation of EAAT2, while promoter hypermethylation is associated with transcriptional silencing (Zschocke et al., 2007). This suggests that the *SLC1A2* promoter hypo- and hypermethylation observed in this study may result in important changes in gene expression, which may significantly alter synaptic function and contribute to the development of BD or the maintenance of concurrent addictive disorders.

Like many epigenetic studies, the results of this investigation do not identify direction of causality; we are unable to ascertain if BD disease pathology results in *SLC1A2* promoter hypermethylation or does *SLC1A2* hypermethylation increase the risk of developing BD. Furthermore, given the limitations of a single cross-sectional analysis, we are not able to clarify if comorbid addictions reduce previous disease associated hypermethylation. Our results demonstrate that BD may be associated with either *SLC1A2* hypermethylation or hypomethylation depending on the presence or absence of concurrent addiction. Longitudinal study by Winokur et al. (1995) provides support for the idea that addiction associated with BD may be for some patients an acquired comorbidity. The primary vs. secondary distinction of substance use disorders in dual diagnosis may be further refined by investigating epigenetic modifications of disease risk genes (Winokur et al., 1995; Guidotti et al., 2013). The peripheral blood which contains different types of cells may be considered to confound DNA methylation analyses. However, study from Philibert et al. (2010) has demonstrated that DNA methylation patterns did not show significant difference between the samples derived from whole blood and lymphoblasts (Philibert et al., 2010). Nevertheless, we certainly cannot rule out the possibility that the alterations in methylation are due to differing composition of cell subtypes in whole blood, which will have to be subject to further investigation (Domschke et al., 2012, 2014; Teroganova et al., 2016). In this study, the *SLC1A2* promoter methylation profile in cellular composition of whole peripheral blood and the blood cell differentiation of the participants were unknown, which might reflect alterations of the blood among diseases and warrant future cell-based investigation.

We analyzed our data taking into consideration a number of covariates, and found that sex (*p* = 0.0365) (**Table 2**) and mood instability (*p* = 0.0329) (**Table 1**) were significantly associated with methylation status of the *SLC1A2* promoter. While previous studies (Chatterjee et al., 2016) have indicated that sex may significantly contribute to differences in promoter methylation status, caution is advised when interpreting the impact of sex in the current study due to the fact that the observed differences are modest and the number of study subjects is relatively small. Although it is tempting to speculate that mood instability may modulate *SLC1A2* promoter methylation, identical reservation is required when interpreting these results, and these findings should be validated with a larger sample. Current smoker status is another univariate predictor of methylation (*p* = 0.0272) (**Table 1**) which is in concert with findings from previous studies that demonstrated the impact of nicotine on DNA demethylation (Satta et al., 2008). For TA cloning and DNA sequencing analysis, there was a statistically significant difference between the BD only group and BD with AA and ND group for the atypical antipsychotics use (*p* = 0.020) (Supplementary Table S1). However, given the small group sample size (*N* = 9 for BD only and *N* = 10 for BD with AA and ND), the comparison was underpowered. Similarly, when larger groups used for HRM-PCR analysis were compared for the atypical antipsychotic use there was even more pronounced difference between the groups (*p* = 0.0016) (**Table 1**). However, when the effect of the use of antipsychotics was analyzed as univariate predictors of methylation temperature no statistically significant effect was found (*p* = 0.5795) (**Table 1**). It is pertinent to mention that study by Veldic et al. (2005) found no significant correlation between intake of atypical antipsychotics and expression levels of DNA-methyltransferase 1 (DNMT1), an enzyme that catalyzes the methylation of cytosine at carbon atoms in position 5 in CpG dinucleotides, and is overexpressed in post-mortem brains and peripheral blood samples of patients with BD and schizophrenia (Veldic et al., 2004, 2005; Zhubi et al., 2009). In fact, in a combined group of patients never treated with antipsychotic drugs and antipsychotic-free patients for at least 3 months before death, DNMT1 mRNA expression was virtually identical to the average levels in patients receiving antipsychotic treatment (Veldic et al., 2005). Taken together, these findings suggest that significant contribution of the antipsychotic use on the level of *SLC1A2* promoter methylation is rather unlikely but also cannot be excluded with the absolute certainty.

Our results demonstrate that the effect of promoter methylation on EAAT2 expression may be site-specific. Several of the CpG sites examined during the course of our study are in close proximity to or overlap with known consensus sequences for the binding of transcription factors such as NF-1, H4TF-2, and SP-1. Although all of these transcription factors primarily act as transcriptional activators, both H4TF-2 and SP-1 may also act as transcriptional repressors (Dailey et al., 1987; Pham N.L. et al., 2004; Pham T.H. et al., 2004). Furthermore, EAAT2 expression and function are known to be regulated by a variety of other transcription factors, such as NFkB, CREB, and YY1, which may also act to positively or negatively influence gene expression (Takahashi et al., 2015; Ayers-Ringler et al., 2016). This suggests that site-specific methylation of the *SLC1A2* promoter may differentially alter the binding affinity of various transcription factors, modulating gene activity according to the relative activating or inhibitory effect of each affected transcription factor. Furthermore, this may provide a mechanistic explanation as to why both hypermethylation and hypomethylation are associated with BD. Although different patterns of DNA methylation may be seen in patients with isolated BD and those with BD with addiction, it is possible that these different patterns of promoter methylation produce similar overall effects on gene transcription. Alternatively, it is possible that different patterns of methylation may promote alternative splicing of the EAAT2 mRNA, which may alter the subcellular localization and functionality of the mature protein and result in similar impairment as a result of hypermethylation in BD or hypomethylation in BD with addiction (Maragakis et al., 2004; Berger et al., 2005; Lauriat and McInnes, 2007). However, to our best knowledge, there have not been any studies investigating the effects of *SLC1A2* promoter methylation on EAAT2 splice variant expression.

While our TA cloning study identifies the differential methylation of three specific CpG sites that are likely candidates for important regulation of *SLC1A2* gene expression, due to the limited number of colonies and subjects, we do not exclude the possibility that other CpG sites may also display changes in methylation if more colonies will be sequenced. However, we were studying the association of methylation with BD only based on *SLC1A2* gene, the sample size for TA cloning and HRM are sufficient to identify the statistical difference for single gene, which is similar as the previous studies (Liu et al., 2010; Shi et al., 2014). Furthermore, we included a conservative method (Bonferroni) for correcting for multiple testing during *post hoc* pairwise comparisons, which may inflate the type I error rate, and should not detract further research of the effects which we found to be marginally non-significant. These results should be validated by additional sequencing and gene expression analyses on samples of human post-mortem brain tissue. It is also important to note that we used BE as a proxy for food addiction rather than a measure of food addiction and while many mechanisms are shared (i.e., reward dysfunction, impulsivity) there are differences unique for addiction (i.e., withdrawal, tolerance) and eating disorder (i.e., dietary restraint, shape/weight concern) frameworks (Schulte et al., 2016). Future carefully designed longitudinal study on a larger sample with serial evaluations of epigenetic changes is warranted in order to add more clarity to understanding of the complicated interactions between addiction and BD. Additional direction to consider for the future studies would be to conduct genome-wide screen of DNA methylation either by Illumina arrays, methyl seq or whole genome bisulfite DNA sequencing, though this was been beyond the scope of the present study.

## Conclusion

To our knowledge, the current study presents the first evidence suggesting that methylation within the *SLC1A2* promoter may be modified by BD and addiction. The results of this study are clinically valuable, as they provide a new possibility of developing epigenetic biomarkers for BD diagnosis and monitoring. While future precision medicine based diagnostic and treatment tools are being developed, astute clinical phenotyping remains a gold standard that can assure high quality individualized treatment for patients with BD.

## Ethics Statement

This study was carried out in accordance with the recommendations of the Mayo Clinic IRB with written informed consent from all subjects. All subjects gave written informed consent in accordance with the Declaration of Helsinki. The protocol was approved by the Mayo Clinic IRB.

## Author Contributions

Y-FJ performed all the data analysis, also helped draft the manuscript and interpreted the findings. Y-FJ, YC, and JA-R contributed to the acquisition of data. JB and JG assisted with data analysis and interpretation of findings. DL, SM, and MF, provided critical revision of the manuscript for important intellectual content. D-SC and MV were responsible for the study concept and design. MV drafted the manuscript. All authors critically reviewed content and approved final version for publication.

## Conflict of Interest Statement

Dr. MF is a consultant (for Mayo Clinic) to Janssen, Mitsubishi Tanabe Pharma Corporation, Myriad, Sunovion, and Teva Pharmaceuticals. None of this funding contributed to work carried out in the present study. Dr. SM is a consultant to or member of the scientific advisory boards of Bracket, MedAvante, F. Hoffmann La Roche, Ltd., Ironshore, Myriad, Naurex, Novo Nordisk, Shire, and Sunovion. She is a principal or co-investigator on studies sponsored by the Agency for Healthcare Research and Quality (AHRQ), Cephalon, Forest, Marriott Foundation, Myriad, National Institute of Mental Health, Naurex, Novo Nordisk, Orexigen Therapeutics, Inc., Shire, and Takeda Pharmaceutical Company, Ltd. She is also an inventor on United States Patent No. 6,323,236 B2, Use of Sulfamate Derivatives for Treating Impulse Control Disorders, and along with the patent’s assignee, University of Cincinnati, Cincinnati, Ohio, has received payments from Johnson and Johnson, which has exclusive rights under the patent. None of this funding contributed to work carried out in the present study. The other authors declare that the research was conducted in the absence of any commercial or financial relationships that could be construed as a potential conflict of interest.

## Footnotes

1 http://www-bimas.cit.nih.gov/molbio/signal/
