# Supplementary material for: Differential SLC1A2 Promoter Methylation in Bipolar Disorder With or Without Addiction
Source: Front Cell Neurosci. 2017 Jul 21;11:217. doi: 10.3389/fncel.2017.00217 (PMC5520464; doi:10.3389/fncel.2017.00217)
Supplement: Supplementary file 1 [file Table_1.DOCX]

**Table S1. Characteristics of participants for TA cloning study from the Mayo Community Biobank and Mayo Clinic Individualized Biobank for Bipolar Disorder.**

| **Variables** | **CL** | **BD only** | **BD+AA+ND** | **p Value** |
| --- | --- | --- | --- | --- |
|  | **N=11** | **N=9** | **N=10** |  |
| Female | 54.6 | 55.6 | 40.0 | 0.7401 |
| Age at enrollment | 44.3±15.4 | 44.6±18.1 | 35.8±11.7 | 0.3562 |
| BMI | - | 28.6±4.2 | 26.7±7.3 | 0.527 |
| CIRS | - | 3.0±3.0 | 3.1±2.8 | 0.742 |
| Mood instability | - | 1.0±1.7 | 1.7±1.8 | 0.428 |
| Rapid cycling | - | 44.4 | 60.0 | 0.656 |
| Increased severity | - | 11.1 | 30.0 | 1.0 |
| Mixed episodes | - | 14.3 | 30.0 | 0.603 |
| Cycle acceleration | - | 22.2 | 30.0 | 1.0 |
| Psychosis | - | 25.0 | 80.0 | 0.054 |
| Anxiety disorder | - | 37.5 | 80.0 | 0.145 |
| Atypical antipsychotics | - | 33.3 | 90.0 | 0.020 |
| Lithium | - | 66.7 | 50.0 | 0.463 |
| Lamotrigine | - | 11.1 | 10.0 | 1.0 |
| Depakote/Valproate | - | 0 | 0 | - |
| Alcohol use (more than 1 monthly) | - | 55.5 | 66.7 | 1.0 |
| Smoked 100 cigarettes | - | 22.2 | 100.0 | 0.029 |
| Current smoker | - | 22.2 | 87.5 | 0.015 |
| Early onset | - | 22.2 | 14.3 | 1.0 |

CL=Normal control subjects, BD=bipolar disorder, BE=binge eating disorder or bulimia nervosa, ND=nicotine dependence, AA=alcohol abuse or dependence. BMI=body mass index, CIRS=cumulative illness rating scale, Early onset=onset of BD before age of 20 years. Age at enrollment, BMI, CIRS, and mood instability index are shown as mean ± standard deviation; remaining variables are shown as a percentage.
