# Supplementary material for: Differential SLC1A2 Promoter Methylation in Bipolar Disorder With or Without Addiction
Source: Front Cell Neurosci. 2017 Jul 21;11:217. doi: 10.3389/fncel.2017.00217 (PMC5520464; doi:10.3389/fncel.2017.00217)
Supplement: Supplementary file 2 [file Table_2.DOCX]

**Table S2. The sequences of bisulfite-specific primers for HRM-PCR and TA cloning.**

| **Primers for HRM-PCR** | | | | |
| --- | --- | --- | --- | --- |
| **Target** | | **Primers sequences** | **Product size** | |
| Amplicon 1  (-1756 to -1465) | | F: 5’-TTTAGTTAGAAGGTGGTGAAGAATTTAAGT-3’ | 291 bp | |
|  |  | R: 5’-AAACAAACAAAACCTCACTTTCCTC-3’ |  |  |
| Amplicon 2  (-782 to -651) | | F: 5’-AATAGTTATTTTTTAGATTTT-3’ | 131 bp | |
|  |  | R: 5’-ATCCCTCCTCTACCATCCCTCC-3’ |  |  |
| **Primers for TA cloning** | | | |  |
| **Target** | **PCR reaction** | **Primers sequences** | **Product size** |  |
| Clone 1 | First PCR  (-1905 to -924) | F: 5’-GGAGGGTTTATTGGGTGTTAG-3’  R: 5’-CTTCAACTCACACTCACCCC-3’ | 981 bp |  |
|  | Nested PCR  (-1833 to -936) | F: 5’-GTATAGAGAAGTGGGAGAAGATAG-3’  R: 5’-CTCACCCCCAAACTCACAAAC-3’ | 897 bp |  |
| Clone 2 | First PCR  (-969 to -14) | F: 5’-GTGGGTGTGTTTGTTTGTGAG-3’  R: 5’-CCCCTCCTCTTCAACACTATC-3’ | 955 bp |  |
|  | Nested PCR  (-953 to -29) | F: 5’-GTGAGTTTGGGGGTGAGTGTGAG-3’  R: 5’-TCTTCAACACTATCCGACAACTAT-3’ | 924 bp |  |
