# Supplementary material for: Differential SLC1A2 Promoter Methylation in Bipolar Disorder With or Without Addiction
Source: Front Cell Neurosci. 2017 Jul 21;11:217. doi: 10.3389/fncel.2017.00217 (PMC5520464; doi:10.3389/fncel.2017.00217)
Supplement: Supplementary file 3 [file Table_3.DOCX]

**Table S3. The methylation level and statistical analysis of each CpG site between groups.**

| **CpG site** | **Group** | | | **P value** | | |
| --- | --- | --- | --- | --- | --- | --- |
|  | **CL**  **(mean ± sem) %** | **BD**  **(mean ± sem) %** | **BD+AA+ND**  **(mean ± sem) %** | **BD *vs* CL** | **BD+AA+ND *vs* CL** | **BD+AA+ND *vs* BD** |
| CpG_2 | 12.73±6.19 | 20.00±10.54 | 12.00±5.33 | 0.5423 | 0.9307 | 0.4943 |
| CpG_3 | 18.18±5.01 | 20.00±10.00 | 8.00±3.27 | 0.8654 | 0.1124 | 0.2496 |
| CpG_4 | 20.00±6.03 | 17.78±6.19 | 24.00±6.53 | 0.8015 | 0.6573 | 0.5010 |
| CpG_5 | 14.55±7.67 | 28.89±4.84 | 20.00±7.89 | 0.1513 | 0.6261 | 0.3632 |
| CpG_6 | 1.82±1.82 | 13.33±4.71 | 0.00±0.00 | **0.0246** * | 0.3535 | **0.0082** * |
| CpG_7 | 5.45±3.90 | 0.00±0.00 | 0.00±0.00 | 0.2242 | 0.1991 | - |
| CpG_8 | 12.73±4.07 | 15.56±7.29 | 10.00±3.33 | 0.7264 | 0.6142 | 0.4826 |
| CpG_9 | 1.82±1.82 | 4.44±2.94 | 4.00±2.67 | 0.4401 | 0.5003 | 0.9119 |
| CpG_10 | 3.64±2.44 | 8.89±4.84 | 6.00±3.06 | 0.3193 | 0.5491 | 0.6128 |
| CpG_11 | 3.64±2.44 | 4.44±4.44 | 6.00±3.06 | 0.8689 | 0.5491 | 0.7727 |
| CpG_12 | 5.45±2.82 | 11.11±4.84 | 12.00±5.33 | 0.3054 | 0.2790 | 0.9040 |
| CpG_13 | 5.45±3.90 | 2.22±2.22 | 4.00±2.67 | 0.5067 | 0.7661 | 0.6196 |
| CpG_14 | 5.45±2.82 | 2.22±2.22 | 2.00±2.00 | 0.3955 | 0.3386 | 0.9414 |
| CpG_15 | 0.00±0.00 | 0.00±0.00 | 2.00±2.00 | - | 0.3062 | 0.3574 |
| CpG_16 | 0.00±0.00 | 2.22±2.22 | 2.00±2.00 | 0.2806 | 0.3062 | 0.9414 |
| CpG_17 | 10.91±5.63 | 8.89±4.84 | 4.00±2.67 | 0.7941 | 0.2970 | 0.3760 |
| CpG_18 | 3.64±2.44 | 2.22±2.22 | 0.00±0.00 | 0.6795 | 0.1724 | 0.3051 |
| CpG_19 | 3.64±3.64 | 2.22±2.22 | 0.00±0.00 | 0.7573 | 0.3535 | 0.3051 |
| CpG_20 | 0.00±0.00 | 0.00±0.00 | 4.00±2.67 | - | 0.1312 | 0.1740 |
| CpG_21 | 5.45±3.90 | 0.00±0.00 | 4.00±2.67 | 0.2242 | 0.7661 | 0.1740 |
| CpG_22 | 1.82±1.82 | 0.00±0.00 | 4.00±2.67 | 0.3800 | 0.5003 | 0.1740 |
| CpG_24 | 0.00±0.00 | 0.00±0.00 | 4.00±4.00 | - | 0.3062 | 0.3574 |
| CpG_26 | 1.82±1.82 | 2.22±2.22 | 2.00±2.00 | 0.8885 | 0.9469 | 0.9414 |
| CpG_27 | 1.82±1.82 | 0.00±0.00 | 4.00±2.67 | 0.3800 | 0.5003 | 0.1740 |
| CpG_30 | 1.82±1.82 | 2.22±2.22 | 0.00±0.00 | 0.8885 | 0.3535 | 0.3051 |
| CpG_31 | 1.82±1.82 | 0.00±0.00 | 0.00±0.00 | 0.3800 | 0.3535 | - |
| CpG_32 | 0.00±0.00 | 0.00±0.00 | 2.00±2.00 | - | 0.3062 | 0.3574 |
| CpG_34 | 1.82±1.82 | 0.00±0.00 | 0.00±0.00 | 0.3800 | 0.3535 | - |
| CpG_35 | 1.82±1.82 | 2.22±2.22 | 2.00±2.00 | 0.8885 | 0.9469 | 0.9414 |
| CpG_36 | 1.82±1.82 | 0.00±0.00 | 0.00±0.00 | 0.3800 | 0.3535 | - |
| CpG_37 | 0.00±0.00 | 2.22±2.22 | 0.00±0.00 | 0.2806 | - | 0.3051 |
| CpG_42 | 0.00±0.00 | 2.22±2.22 | 0.00±0.00 | 0.2806 | - | 0.3051 |
| CpG_43 | 0.00±0.00 | 0.00±0.00 | 2.00±2.00 | - | 0.3062 | 0.3574 |
| CpG_44 | 0.00±0.00 | 0.00±0.00 | 2.00±2.00 | - | 0.3062 | 0.3574 |
| CpG_45 | 5.45±3.90 | 2.22±2.22 | 4.00±2.67 | 0.5067 | 0.7661 | 0.6196 |
| CpG_46 | 3.64±2.44 | 2.22±2.22 | 2.00±2.00 | 0.6795 | 0.6142 | 0.9414 |
| CpG_47 | 1.82±1.82 | 4.44±2.94 | 2.00±2.00 | 0.4401 | 0.9469 | 0.4934 |
| CpG_48 | 7.27±4.07 | 11.11±4.84 | 0.00±0.00 | 0.5483 | 0.1051 | **0.0267** * |
| CpG_49 | 5.45±2.82 | 2.22±2.22 | 4.00±2.67 | 0.3955 | 0.7132 | 0.6196 |
| CpG_50 | 0.00±0.00 | 0.00±0.00 | 4.00±2.67 | - | 0.1312 | 0.1740 |
| CpG_51 | 0.00±0.00 | 4.44±2.94 | 0.00±0.00 | 0.1099 | - | 0.1283 |
| CpG_52 | 3.64±2.44 | 0.00±0.00 | 0.00±0.00 | 0.1964 | 0.1724 | - |
| CpG_53 | 5.45±3.90 | 0.00±0.00 | 0.00±0.00 | 0.2242 | 0.1991 | - |
| CpG_54 | 0.00±0.00 | 0.00±0.00 | 0.00±0.00 | 0.3800 | 0.3535 | - |
| CpG_55 | 3.64±2.44 | 0.00±0.00 | 2.00±2.00 | 0.1964 | 0.6142 | 0.3574 |
| CpG_58 | 3.64±2.44 | 2.22±2.22 | 0.00±0.00 | 0.6795 | 0.1724 | 0.3051 |
| CpG_59 | 1.82±1.82 | 0.00±0.00 | 0.00±0.00 | 0.3800 | 0.3535 | - |
| CpG_60 | 5.45±5.45 | 2.22±2.22 | 2.00±2.00 | 0.6186 | 0.5742 | 0.9414 |
| CpG_61 | 3.64±2.44 | 4.44±2.94 | 0.00±0.00 | 0.8334 | 0.1724 | 0.1283 |
| CpG_62 | 1.82±1.82 | 2.22±2.22 | 0.00±0.00 | 0.8885 | 0.3535 | 0.3051 |
| CpG_63 | 9.09±5.63 | 2.22±2.22 | 4.00±2.67 | 0.3097 | 0.4392 | 0.6196 |
| CpG_64 | 10.91±5.63 | 0.00±0.00 | 2.00±2.00 | 0.0984 | 0.1683 | 0.3574 |
| CpG_65 | 7.27±5.57 | 2.22±2.22 | 0.00±0.00 | 0.4478 | 0.2297 | 0.3051 |
| CpG_66 | 1.82±1.82 | 0.00±0.00 | 0.00±0.00 | 0.3800 | 0.3535 | - |
| CpG_67 | 9.09±5.63 | 0.00±0.00 | 2.00±2.00 | 0.1636 | 0.2685 | 0.3574 |
| CpG_70 | 0.00±0.00 | 2.22±2.22 | 0.00±0.00 | 0.2806 | - | 0.3051 |
| CpG_71 | 3.64±2.44 | 6.67±4.71 | 0.00±0.00 | 0.5544 | 0.1724 | 0.1531 |
| CpG_72 | 0.00±0.00 | 0.00±0.00 | 2.00±2.00 | - | 0.3062 | 0.3574 |
| CpG_75 | 5.45±5.45 | 0.00±0.00 | 4.00±2.67 | 0.3800 | 0.8190 | 0.1740 |
| CpG_76 | 1.82±1.82 | 2.22±2.22 | 0.00±0.00 | 0.8885 | 0.3535 | 0.3051 |
| CpG_77 | 3.64±2.44 | 0.00±0.00 | 2.00±2.00 | 0.1964 | 0.6142 | 0.3574 |
| CpG_78 | 0.00±0.00 | 2.22±2.22 | 0.00±0.00 | 0.2806 | - | 0.3051 |
| CpG_81 | 0.00±0.00 | 0.00±0.00 | 4.00±2.67 | - | 0.1312 | 0.1740 |
| CpG_82 | 0.00±0.00 | 0.00±0.00 | 2.00±2.00 | - | 0.3062 | 0.3574 |
| CpG_83 | 0.00±0.00 | 0.00±0.00 | 2.00±2.00 | - | 0.3062 | 0.3574 |
| CpG_85 | 0.00±0.00 | 0.00±0.00 | 2.00±2.00 | - | 0.3062 | 0.3574 |
| CpG_86 | 0.00±0.00 | 0.00±0.00 | 4.00±2.67 | - | 0.1312 | 0.1740 |
| CpG_87 | 1.82±1.82 | 0.00±0.00 | 0.00±0.00 | 0.3800 | 0.3535 | - |
| CpG_88 | 1.82±1.82 | 0.00±0.00 | 0.00±0.00 | 0.3800 | 0.3535 | - |
| CpG_89 | 0.00±0.00 | 2.22±2.22 | 6.00±4.27 | 0.2806 | 0.1558 | 0.4583 |
| CpG_91 | 1.82±1.82 | 0.00±0.00 | 0.00±0.00 | 0.3800 | 0.3535 | - |
| CpG_92 | 0.00±0.00 | 0.00±0.00 | 2.00±2.00 | - | 0.3062 | 0.3574 |
| CpG_93 | 0.00±0.00 | 2.22±2.22 | 4.00±2.67 | 0.2806 | 0.1312 | 0.6196 |
| CpG_94 | 1.82±1.82 | 2.22±2.22 | 2.00±2.00 | 0.8885 | 0.9469 | 0.9414 |
| CpG_96 | 1.82±1.82 | 2.22±2.22 | 6.00±4.27 | 0.8885 | 0.3630 | 0.4583 |
| CpG_97 | 0.00±0.00 | 0.00±0.00 | 2.00±2.00 | - | 0.3062 | 0.3574 |
| CpG_98 | 1.82±1.82 | 4.44±2.94 | 2.00±2.00 | 0.4401 | 0.9469 | 0.4934 |
| CpG_100 | 0.00±0.00 | 2.22±2.22 | 0.00±0.00 | 0.2806 | - | 0.3051 |
| CpG_101 | 1.82±1.82 | 2.22±2.22 | 6.00±3.06 | 0.8885 | 0.2439 | 0.3406 |
| CpG_104 | 3.64±2.44 | 0.00±0.00 | 2.00±2.00 | 0.1964 | 0.6142 | 0.3574 |
| CpG_105 | 0.00±0.00 | 0.00±0.00 | 2.00±2.00 | - | 0.3062 | 0.3574 |
| CpG_106 | 0.00±0.00 | 2.22±2.22 | 0.00±0.00 | 0.2806 | - | 0.3051 |
| CpG_107 | 1.82±1.82 | 0.00±0.00 | 2.00±2.00 | 0.3800 | 0.9469 | 0.3574 |
| CpG_112 | 1.82±1.82 | 4.44±2.94 | 0.00±0.00 | 0.4401 | 0.3535 | 0.1283 |
| CpG_113 | 0.00±0.00 | 0.00±0.00 | 2.00±2.00 | - | 0.3062 | 0.3574 |
| CpG_114 | 1.82±1.82 | 0.00±0.00 | 2.00±2.00 | 0.3800 | 0.9469 | 0.3574 |
| CpG_115 | 0.00±0.00 | 2.22±2.22 | 0.00±0.00 | 0.2806 | - | 0.3051 |
| CpG_116 | 0.00±0.00 | 2.22±2.22 | 0.00±0.00 | 0.2806 | - | 0.3051 |
| CpG_118 | 3.64±3.64 | 0.00±0.00 | 0.00±0.00 | 0.3800 | 0.3535 | - |
| CpG_119 | 3.64±2.44 | 0.00±0.00 | 2.00±2.00 | 0.1964 | 0.6142 | 0.3574 |
| CpG_120 | 1.82±1.82 | 0.00±0.00 | 0.00±0.00 | 0.3800 | 0.3535 | - |
| CpG_121 | 3.64±2.44 | 0.00±0.00 | 0.00±0.00 | 0.1964 | 0.1724 | - |
| CpG_122 | 1.82±1.82 | 2.22±2.22 | 0.00±0.00 | 0.8885 | 0.3535 | 0.3051 |
| CpG_123 | 1.82±1.82 | 0.00±0.00 | 0.00±0.00 | 0.3800 | 0.3535 | - |
| CpG_124 | 0.00±0.00 | 0.00±0.00 | 2.00±2.00 | - | 0.3062 | 0.3574 |
| CpG_125 | 1.82±1.82 | 0.00±0.00 | 0.00±0.00 | 0.3800 | 0.3535 | - |
| CpG_126 | 0.00±0.00 | 0.00±0.00 | 2.00±2.00 | - | 0.3062 | 0.3574 |
| CpG_127 | 1.82±1.82 | 0.00±0.00 | 2.00±2.00 | 0.3800 | 0.9469 | 0.3574 |
| CpG_128 | 1.82±1.82 | 0.00±0.00 | 0.00±0.00 | 0.3800 | 0.3535 | - |
| CpG_129 | 7.27±5.57 | 6.67±3.33 | 2.00±2.00 | 0.9308 | 0.4028 | 0.2356 |
| CpG_131 | 3.64±2.44 | 0.00±0.00 | 0.00±0.00 | 0.1964 | 0.1724 | - |
| CpG_135 | 0.00±0.00 | 0.00±0.00 | 2.00±2.00 | - | 0.3062 | 0.3574 |
| CpG_136 | 0.00±0.00 | 4.44±2.94 | 2.00±2.00 | 0.1099 | 0.3062 | 0.4934 |
| CpG_137 | 9.09±5.63 | 4.44±2.94 | 2.00±2.00 | 0.5028 | 0.2685 | 0.4934 |
| CpG_138 | 1.82±1.82 | 0.00±0.00 | 2.00±2.00 | 0.3800 | 0.9469 | 0.3574 |
| CpG_139 | 1.82±1.82 | 0.00±0.00 | 0.00±0.00 | 0.3800 | 0.3535 | - |
| CpG_140 | 1.82±1.82 | 2.22±2.22 | 0.00±0.00 | 0.8885 | 0.3535 | 0.3051 |
| CpG_141 | 1.82±1.82 | 0.00±0.00 | 0.00±0.00 | 0.3800 | 0.3535 | - |
| CpG_142 | 1.82±1.82 | 0.00±0.00 | 0.00±0.00 | 0.3800 | 0.3535 | - |
| CpG_144 | 1.82±1.82 | 0.00±0.00 | 0.00±0.00 | 0.3800 | 0.3535 | - |
| CpG_146 | 0.00±0.00 | 2.22±2.22 | 0.00±0.00 | 0.2806 | - | 0.3051 |
| CpG_149 | 1.82±1.82 | 0.00±0.00 | 0.00±0.00 | 0.3800 | 0.3535 | - |
| CpG_150 | 0.00±0.00 | 0.00±0.00 | 2.00±2.00 | - | 0.3062 | 0.3574 |
| CpG_152 | 0.00±0.00 | 0.00±0.00 | 6.00±4.27 | - | 0.1558 | 0.2012 |
| CpG_154 | 7.27±7.27 | 0.00±0.00 | 2.00±2.00 | 0.3800 | 0.5114 | 0.3574 |
| CpG_155 | 0.00±0.00 | 0.00±0.00 | 2.00±2.00 | - | 0.3062 | 0.3574 |
| CpG_156 | 98.18±1.82 | 95.56±2.94 | 77.78±8.03 | 0.4401 | **0.0184** * | 0.0646 |

The unmethylated CpG sites were not shown in here; * and bold letters indicate the statistically significant difference (p < 0.05).
